# The DASH Diet and Cardiometabolic Health and Chronic Kidney Disease: A Narrative Review of the Evidence in East Asian Countries

**DOI:** 10.3390/nu13030984

**Published:** 2021-03-18

**Authors:** Yazhen Song, Andrea J. Lobene, Yanfang Wang, Kathleen M. Hill Gallant

**Affiliations:** 1 Peking University Clinical Research Institute, Beijing 100191, China; sophiasong0829@gmail.com (Y.S.); yanfang1225@gmail.com (Y.W.); 2 Department of Nutrition Science, Purdue University, West Lafayette, IN 47907, USA; hillkm@umn.edu; 3 Department of Kinesiology and Applied Physiology, University of Delaware, Newark, DE 19713, USA; 4 Department of Food Science and Nutrition, University of Minnesota, St. Paul, MN 55108, USA

**Keywords:** DASH diet, cardiovascular disease, type 2 diabetes, chronic kidney disease, cardiometabolic health, East Asia, China, Japan, South Korea

## Abstract

The rising incidence of cardiometabolic diseases and chronic kidney disease (CKD) is a leading public health problem in East Asia. Diet is an important modifiable risk factor; thus, adopting a healthy diet such as the Dietary Approaches to Stop Hypertension (DASH) diet may help combat these chronic diseases. The DASH diet was originally developed in a U.S. population, and East Asia is demographically and culturally different from the U.S. Therefore, it is important to examine the evidence regarding the DASH diet and chronic disease in this unique population. This narrative review summarizes the evidence on the DASH diet and cardiometabolic health and CKD in East Asia. Culturally-modified DASH diets have been developed in some East Asian countries. Studies suggest the DASH diet is effective at lowering blood pressure in this population, though the long-term benefits remain unclear. Evidence also suggests the DASH diet may reduce the risk of type 2 diabetes and metabolic syndrome. Further research indicates the DASH diet and its components may reduce CKD risk. However, recommending the DASH diet in those who already have CKD is controversial, as it conflicts with current CKD dietary guidelines, especially in advanced CKD. Notably, current intakes in the general population differ from the DASH dietary pattern, suggesting public health efforts would be needed to encourage adoption of the DASH diet.

## 1. Introduction

The increasing incidence of chronic diseases like cardiovascular disease (CVD), diabetes, and chronic kidney disease (CKD) has become a significant health problem in Asia [1,2]. In China, CVD has become the top cause of death, with approximately 290 million prevalent cases during 2016 [3]. East Asian countries also have a high prevalence of type 2 diabetes (China 9.6%, Taiwan 9.8%, Japan 7.6%, South Korea 8.9%) with China and Japan among the top 10 countries globally for number of people with type 2 diabetes [4]. Among East Asian patients with hypertension and type 2 diabetes, 40–70% have micro or macroalbuminuria, which is an indicator of kidney damage and risk factor for CVD mortality [5]. Hypertension and diabetes are the most common underlying etiologies for CKD [6], and patients often present with two or more of these conditions together. Indeed, in China, the prevalence of hypertension in CKD patients is 78.4% [7]. Both high-income and middle-income countries in East Asia face the challenge of increasing CKD prevalence, partially driven by an aging population [8], which is especially challenging in developing countries [9]. In China, the largest developing country in the world, the prevalence of CKD is 10.8% or about 119.5 million people based on the results of a nationally representative survey [10]. However, only 12.5% of those with CKD in China are aware of their condition [10,11]. Given these data, it is clear that developing interventions to target blood pressure (BP) regulation and blood glucose control are important for preventing chronic diseases such as CVD, type 2 diabetes, and CKD. Dietary factors are important contributors to the incidence of these chronic diseases. The new Global Burden of Disease Study 1990–2017 found that East Asia has the highest age-standardized rates of diet-related CVD deaths and disability-adjusted life years (DALYs) around the world [12]. This same study found that a high intake of sodium was the leading dietary risk factor for deaths and DALYs in East Asia, and other dietary risk factors in China included low intake of whole grains and fruit in China [12]. Indeed, high BP caused by salt consumption is shown to be a major contributor to CVD in China [13], South Korea [14], and Japan [15], as well as other East Asian countries [16]. These findings suggest that improving dietary intakes in East Asian countries could be an important strategy for combating chronic disease.

When considering dietary recommendations for reducing chronic disease incidence and risk in East Asia, one intervention to consider is the Dietary Approaches to Stop Hypertension (DASH) diet. The original DASH trials were conducted in the United States [17,18], and since then the DASH diet and its relationship to chronic disease has been studied all over the world with largely positive results [19,20,21,22,23]. However, each region and country has unique demographic and cultural characteristics that may impact the efficacy and effectiveness of the DASH diet. For example, East Asians develop type 2 diabetes at a younger age and a lower BMI compared to Caucasians, and these patients are more likely to experience a stroke or develop renal complications [24]. In addition, East Asians have different beliefs and attitudes about healthy eating compared to the U.S. [25] Thus, it is important to review the evidence within specific cultural or regional populations. While studies have examined the DASH diet and its relationship to cardiometabolic health and CKD in East Asia, the evidence has not been reviewed or synthesized previously. Thus, the purpose of this narrative review is to summarize and discuss the available evidence regarding the DASH diet, as well as its components, as it relates to cardiometabolic disease and CKD risk and progression in East Asia.

## 2. Characteristics of the DASH Diet and the Original Aim of the DASH Trials

The original DASH trial was the first study to investigate the effect of consuming an overall healthy dietary pattern on BP using a randomized controlled feeding study [26]. While the control dietary pattern was designed to reflect the usual American diet, the intervention dietary patterns were strategically designed to contain nutrients previously shown to be beneficial for BP reduction. The “ideal” or “combination” dietary pattern, which later became known simply as the DASH diet, emphasized vegetables, fruits, low-fat dairy products, whole grains, fish, poultry, and nuts, and was low in red meat, sweets, and sugar-sweetened beverages [17]. The trial enrolled 459 healthy adults with normal to elevated BP, who followed their assigned diet for eight weeks [26]. The results showed that the DASH diet reduced BP to a greater extent than the control diet and the intermediate “fruits and vegetables” diet. These findings indicate that adopting an overall healthy dietary pattern can effectively reduce BP.

The original DASH trial did not address the effect of sodium intake on BP, as all three study diets contained similar levels of sodium (~3000 mg/d) [26]. Given the body of evidence suggesting lowering sodium intake may reduce BP, the same research group conducted a second controlled feeding study aimed at investigating the effect of the DASH diet combined with reduced sodium intake on BP [27]. Within the context of the DASH and control (i.e., usual American) dietary patterns, the researchers investigated three different sodium levels: “high” (150 mmol/d or ~3500 mg/d), “intermediate” (100 mmol/d or ~2300 mg/d), and “low” (50 mmol/d or ~1200 mg/d) [18]. The trial enrolled 412 adults with elevated BP, who were randomly assigned to one of the two dietary patterns (DASH or control). Participants consumed their assigned dietary pattern at each of the three sodium levels, in randomized order, for 30 days each. The results showed that, within both dietary patterns, lower sodium intake was effective at lowering BP. However, the greatest benefit to BP was observed when the DASH diet was combined with lowest sodium intake (1200 mg/d). Taken together, the findings from both trials suggest that adopting a DASH dietary pattern, especially combined with low sodium intake, is an effective strategy for reducing BP.

## 3. The DASH Diet Compared to Usual Dietary Intakes in East Asia

Since the original trials were published, the DASH diet has been examined and tested in many countries throughout the world with largely positive results. However, because the DASH diet was developed for a U.S. population, it is important to understand how the cultural beliefs and dietary practices of East Asian differ from the U.S. before examining the evidence regarding the DASH diet in this region specifically. For example, in a recent study examining beliefs about healthy diets in young adults in the U.S. and China, participants from both countries agreed on the importance of fruits and vegetables; however, Chinese participants believed meat and foods high in fat and salt should be limited [25]. In addition, Chinese participants in this study emphasized timing of meals, portion sizes, and the types of foods consumed at each eating occasion as factors of a healthy diet. In addition to differences in beliefs regarding food, the U.S. and East Asia differ in the types of foods commonly consumed. Indeed, soy and soy products are regularly consumed in East Asian countries, but are eaten less frequently in the U.S., and evidence suggests this increased consumption of soy products may confer a benefit in terms of chronic disease incidence and mortality [28]. However, Asian diets are becoming increasingly more Westernized, with increased consumption of wheat, high protein and energy-dense foods, potatoes, and dairy, to name a few [29]. All of these dietary factors provide context for understanding how the DASH diet may be adopted and studied in this region.

It is also important to understand how usual dietary intakes in East Asia compare to the original DASH diet guidelines. Comparisons between the DASH diet recommendations [17,18] and usual dietary intakes of adults in major East Asian countries, adapted from Zhang et al. [30], are summarized in Table 1. Nutrient intakes, especially mineral and fiber intakes, are different compared with the DASH diet, which may be partially attributed to increased Westernization of East Asian diets. The traditional Chinese diet has plenty of grains and vegetables [31]. Due in part to economic development and urbanization, Chinese people have shifted from mostly eating at home to now more frequently dining out; as a result, the fast food industry has grown rapidly [32] and fresh vegetable and fiber intakes have decreased in those years [31,33]. Indeed, according to the Global Burden of Disease study, the average intakes of vegetables and fruits in China are 350 g/d [34] and 80 g/d [12], respectively. In Japan, evidence suggests dietary patterns characterized by plant foods (e.g., fruits and vegetables) and fish have decreased over the past few decades, whereas dietary patterns characterized by high intakes of sugar and red and processed meat have increased [35]. In addition, older Japanese adults tend to adhere more closely to the traditional Japanese diet whereas younger Japanese adults do not, resulting in lower intakes of fruits, vegetables, legumes, fish, and dairy and higher intakes of meats [36]. Though the data are not shown in Table 1, similar trends are observed in South Korea. Indeed, the average sodium intake in South Korea is nearly 4000 mg/d [37]. In addition, while fruit and vegetable intake has increased over the past several decades, intake of meat and fat has also increased, and intakes of micronutrients such as calcium and potassium remained steady but insufficient [38]. Together, these data suggest that usual dietary intakes in East Asia do not align with DASH diet guidelines.

## 4. The DASH Diet and Cardiometabolic Health in East Asia

As discussed in the previous section, it is important to review the evidence regarding the DASH diet and various health outcomes within specific cultural or regional populations to fully understand its effectiveness. The following sections will review the evidence regarding the relationship between the DASH diet and cardiometabolic health in East Asian populations. The papers reviewed in this section are summarized in Supplemental Table S1.

### 4.1. The DASH Diet and Cardiovascular Disease

The DASH diet was originally developed for a U.S. population; thus, culturally-modified DASH diets have been developed for both Japan [39,40] and South Korea [41,42]. A key feature of both the DASH-Japan Ube Modified diet Program (DASH-JUMP) and the Korean DASH diet (K-DASH) is the incorporation of staple foods, traditional dishes, and other dietary practices from their respective countries into the culturally-modified DASH diet. As with the original DASH trials, the DASH-JUMP diet was investigated in the context of a controlled feeding study, in which participants were provided all food throughout the course of the intervention [39]. Thus, detailed nutrient composition data on the study menus have been published, and are compared to the original DASH diet in Table 2. The original DASH diet and DASH-JUMP have similar nutrient compositions, with the most noticeable difference being the macronutrient distribution and the sodium levels. K-DASH, however, was originally investigated in a free-living context, in which participants were not given food but rather were educated on how to adopt the culturally-modified DASH diet [41]. Thus, detailed study menus and nutrient content for K-DASH have not been reported and could not be included in Table 2.

Both DASH-JUMP and K-DASH have been investigated to determine the effect on CVD risk factors. In the DASH-JUMP study, 58 adults with elevated BP consumed the DASH-JUMP diet for 2 months, then consumed their usual diets for 4 months. The study found that consuming the DASH-JUMP diet resulted in significant decreases in systolic and diastolic BP, which significantly increased after participants returned to their usual dietary intake. The K-DASH diet was originally tested in Korean Americans with elevated BP [41]; the 10-week pilot study found that providing structured education on this culturally adapted DASH diet resulted in significant reductions in ambulatory BP. A later study in Korea explored the effect of an 8-week lifestyle modification intervention, which included the K-DASH diet and exercise, in adults with elevated BP, and observed significant reductions in ambulatory BP [42]. These studies demonstrate the BP benefits of culturally-modified DASH diet interventions in both clinical and free-living settings. However, these studies were relatively short-term. One study from Hong Kong found that providing a one-time counselling session on the DASH diet to patients with newly diagnosed hypertension did not result in significant improvements in cardiovascular risk factors, including blood pressure, BMI, and lipid profile, at 6 or 12 months compared to the usual standard of care [43]. The effect of more frequent DASH diet education/counseling on CVD risk in East Asian populations has not been explored.

In addition to the intervention studies cited above, observational studies from East Asian countries have explored the link between the DASH diet and CVD. Indeed, a Chinese prospective cohort study found that adopting healthy lifestyle factors, including a DASH-style diet, was associated with a lower risk of hypertension [44]. Another prospective study in Taiwanese adults found that adherence to a DASH-style diet was inversely associated with change in systolic BP and risk of stroke [45]. When examining the components of the DASH diet, this Taiwanese study also found that both dairy and calcium intake were inversely associated with change in systolic BP. An additional prospective cohort study in Singaporean Chinese adults found that greater adherence to a DASH-style diet was associated with a decreased risk of CVD mortality [46]. In this study, further examination of the DASH diet components found that increased intake of vegetables, fruits, nuts, fiber, and n-3 fatty acids, and decreased intake of red meat were associated with decreased risk of CVD mortality. Additional research from the same cohort study found that greater adherence to the DASH diet was associated with lower risk of coronary artery disease or stroke mortality [47]. Together, these studies suggest DASH diet adherence is inversely associated with CVD risk in East Asian populations.

### 4.2. The DASH Diet and Type 2 Diabetes and Other Metabolic Disturbances

Studies in East Asian populations have also explored the link between the DASH diet and type 2 diabetes. A prospective study using data from the Singapore Chinese Health Study found that greater adherence to a DASH-style diet was associated with a 29% lower risk of developing type 2 diabetes [48]. One Korean intervention study explored the glycemic benefits of a DASH-style diet [49]. In this study, 60 adults with type 2 diabetes were randomly assigned to receive either a DASH-style diet plan with 2 pre-portioned meals per day, a Food Exchange diet plan with 2 pre-portioned meals per day, or a Food Exchange diet plan with no pre-portioned meals for 12 weeks. They observed significant reductions in glycated hemoglobin (HbA1C) in the group assigned to the DASH-style diet compared to the other intervention groups. Together, these studies provide preliminary evidence on the link between the DASH diet and type 2 diabetes risk and management in East Asian populations, though further research is needed.

The relationship between DASH diet adherence and other metabolic disturbances has been explored in East Asian populations. One such disturbance is metabolic syndrome, which is a cluster of factors that are associated with increased risk of cardiometabolic disease, including dyslipidemia, elevated BP, elevated blood glucose, and larger waist circumference [50]. A cross-sectional study using Korean National Health and Nutrition Examination Survey (KNHANES) data found that, in postmenopausal women without diabetes, better adherence to the DASH diet was associated with lower prevalence of metabolic syndrome [51]. Another cross-sectional study using data from the National Health and Nutrition Survey, Japan found that better adherence to the DASH diet was inversely associated with metabolic risk factors including waist circumference, total cholesterol, LDL cholesterol, and BMI [52]. This observational evidence suggests a beneficial effect of the DASH diet for metabolic syndrome, and findings from intervention studies seem to agree [53,54]. Indeed, one randomized-controlled trial investigated the effect of an 8-week individualized DASH diet education intervention plus omega-3 fatty acid supplementation on metabolic syndrome parameters in elderly South Korean women [53]. After 8 weeks, DASH diet adherence was significantly higher, and LDL and triglyceride levels were significantly lower in the experimental group, but not in the control group, who only received one DASH diet education session and did not receive omega-3 supplements. Another risk factor for cardiometabolic disease, as well as CKD, is hyperuricemia. Dietary factors are linked to hyperuricemia, and while the evidence is scarce, one observational study from China found that greater adherence to a DASH-style diet is associated with a reduced risk of hyperuricemia [54]. Together, these studies suggest that the DASH diet may be beneficial for metabolic disturbances associated with cardiometabolic disease.

## 5. The DASH Diet and Chronic Kidney Disease

To our knowledge, only one study from East Asia has explored the relationship between the DASH diet and CKD risk. Indeed, a cross-sectional study using KNHANES data found that elderly adults with greater adherence to a DASH-style diet had lower odds of having CKD [55]. In addition, as mentioned in the previous section, a study from China found that adherence to a DASH-style diet is associated with a reduced risk of hyperuricemia, which itself is a risk factor for CKD [54]. No study from East Asia has explored the relationship between the DASH diet and CKD progression or CKD-related complications.

Previous reviews from other countries have acknowledged that recommending the DASH dietary pattern in CKD is controversial due to concerns regarding protein, potassium, and phosphorus intake, particularly in advanced CKD [56]. Therefore, closer examination of the components of the DASH diet (i.e., specific nutrients or food groups emphasized in the DASH diet) may be warranted to explore strategies for developing a modified DASH diet for individuals with CKD. As discussed previously, the DASH dietary pattern was carefully designed to increase nutrients considered to be beneficial for BP (i.e., calcium, potassium, magnesium, protein, and fiber), and to limit nutrients thought to negatively affect BP (i.e., sodium and saturated fat) [26,27]. When translated into foods, the DASH diet emphasizes the intake of fruits and vegetables; low-fat dairy products; whole grains; lean meat, poultry, and fish; and nuts, seeds, and legumes, and minimizes the intake of full-fat dairy; high-fat meats; and products with added sugars [57]. Below, we review the evidence regarding the relationships between DASH diet components and CKD risk and CKD complications in East Asian populations. The papers reviewed in this section are summarized in Supplemental Table S1.

### 5.1. DASH Diet Components and the Risk of Developing CKD

Minerals, particularly sodium and potassium, are important components of the DASH dietary pattern. In the DASH diet, sodium intake is limited due to the well-documented effects of excess sodium intake on BP and CVD risk [27]. Given that hypertension is the most common underlying etiology for CKD [6], sodium intake is an important consideration for CKD risk. A prospective cohort study from South Korea found that, in participants with hypertension, both low and high sodium intakes were associated with an increased risk of developing CKD [58]. However, in participants without hypertension, there was no difference in CKD risk based on sodium intake level. These findings may suggest that sodium intake is a risk factor for CKD in those who are already at increased risk of developing CKD, though clinical studies are needed to confirm this. The only intervention study from East Asia examining sodium reduction and CKD risk comes from an 18-month cluster-randomized trial in China [59]. In this study, 120 villages were randomized to receive either a sodium reduction program, which included community health education and providing access to a potassium-based salt substitute, or the control. They found that, compared to control, participants in the sodium reduction program had lower urinary albumin-to-creatinine ratio and lower odds of albuminuria, both of which are indicators of kidney function [59]. Contrary to sodium, potassium intake is emphasized in the DASH diet. A prospective cohort study in South Korean adults with impaired kidney function found that a higher potassium intake was associated with a decreased risk of CKD development and less estimated glomerular filtration rate (eGFR) decline, but only in participants with hypertension [60]. These findings are corroborated by a cross-sectional study from South Korea, which found that low potassium intake was associated with increased odds of advanced stage CKD, though only in participants with hypertension [61]. Phosphorus, though not included in the DASH diet guidelines, is another mineral that may be higher in this diet due to increased intakes of dairy, whole grains, and lean protein sources. The same cross-sectional study from South Korea also examined phosphorus intake and similarly found that lower phosphorus intake was associated with increased risk of advanced stage CKD in participants with hypertension [61]. It should be noted that dietary guidance typically recommends that patients with CKD reduce their intakes of potassium and phosphorus in order to avoid CKD progression and further complications. Further research, including clinical evidence, is needed to determine potassium and phosphorus recommendations for patients at risk of CKD, particularly in the context of a DASH-style dietary pattern.

Other nutrients and food groups that are emphasized in the DASH diet may have implications for CKD risk. Fruits and vegetables are an important part of the DASH diet, and evidence suggests that they could be beneficial for preventing CKD. A South Korean prospective cohort study found that, in participants with normal kidney function at baseline, a diet rich in fruits and vegetables was associated with a decreased risk of incident CKD and proteinuria [62]. This study found that a diet high in fruits and vegetables had a lower dietary acid load, as assessed by estimated net endogenous acid production (eNEAP), and postulate this may contribute to the reduction in CKD risk [62]. The eNEAP is a calculated ratio of dietary protein to potassium; higher potassium intake results in a lower eNEAP, and higher protein intake results in a higher eNEAP. As with potassium and phosphorus, dietary guidelines recommend patients with CKD reduce their protein intake. One cross-sectional study from Japan found that higher protein (total, animal, vegetable) intake was associated with a higher GFR in both men and women, and a lower risk of CKD in women [63]. While these results suggest that higher protein intake may be protective against CKD, these results should be interpreted with caution, as they may be influenced by reverse causality (i.e., patients reduce their protein intake after being diagnosed with CKD upon recommendations from their doctor). The source of protein, especially different animal proteins, could also play a role in CKD risk. A prospective cohort study of a Chinese population in Singapore found that consumption of red meat, which tends to be higher in saturated fat, was positively associated with end-stage kidney disease (ESKD); however, no such association was found between leaner sources of protein, including fish, eggs, and poultry, with ESKD risk [64]. Further research is needed to fully elucidate the relationship between individual DASH diet components and CKD risk in an East Asian population.

### 5.2. The DASH Diet and Its Components in Diagnosed CKD

To our knowledge, no study from East Asia has investigated the DASH diet in patients who already have CKD. However, studies have explored the effect of components of the DASH diet, including minerals, fruits, and vegetables, in patients with CKD. As discussed previously, reducing sodium intake can help lower BP and ultimately reduce CKD risk. In those who already have CKD, reducing sodium intake seems to help with lowering BP and slowing the progression of kidney damage. A cross-sectional study in South Korean patients with all stages of CKD found that those with lower 24-h urinary sodium excretion, an indicator of sodium intake, more often achieved appropriate BP control [65]. An intervention study from China similarly found that lower sodium intake was associated with a higher rate of BP control in patients with non-dialysis hypertensive CKD [66]. This Chinese publication also included a small dietary sodium restriction study conducted at the same clinic, which found that a 7-day sodium restriction intervention in patients with immunoglobulin A nephropathy (IgAN) resulted in a significant decrease in BP as well as proteinuria [66]. Together, these findings suggest that reducing sodium intake in patients with CKD may help to achieve BP control and reduce kidney damage. However, as one Chinese study found, reducing sodium intake may be difficult for patients with non-dialysis dependent CKD [67]. While most patients in this study indicated they were aware of the need to restrict dietary sodium, reported barriers to achieving dietary sodium reduction largely included a lack of knowledge, especially regarding condiment use in cooking. This suggests that East Asian patients with CKD could benefit from reducing their sodium intake, though more public health efforts are needed to help these patients make dietary changes.

Other mineral components of the DASH diet are important to consider in terms of CKD progression. While potassium is associated with CKD prevention, the effect of potassium intake in patients with CKD is controversial, as hyperkalemia is a concern in these patients. However, evidence on the effect of potassium reduction in CKD is mixed. A South Korean cohort study in patients with non-dialysis CKD stages 1–5 found that lower urinary potassium excretion, an indicator of potassium intake, is associated with a higher risk of CKD progression, defined as a ≥50% decrease in eGFR and onset of ESKD [68]. A separate South Korean cohort study in patients with non-dialysis CKD stages 1–5 found that a higher urinary Na/K ratio (i.e., relatively higher sodium intake and relatively lower potassium intake) was associated with a greater risk of renal outcomes, defined as a 50% reduction in eGFR or initiation of renal replacement therapy [69]. Together, these studies suggest that a higher potassium intake may be beneficial for East Asians with CKD. However, more evidence is needed to truly understand the risks and benefits of potassium intake in patients with CKD, especially in patients with advanced CKD. Magnesium is another mineral emphasized in the DASH diet because of the demonstrated inverse relationship between magnesium intake and BP [26]. One randomized controlled trial in Japanese patients with CKD stages 3–4 examined the effect of 2 years of magnesium oxide supplementation on coronary artery calcification, which is a risk factor cardiovascular events and mortality in CKD [70]. They found that supplementation of magnesium oxide resulted in significantly lower coronary artery calcification progression compared to the control group. These findings suggest that the effect of increased magnesium intake on cardiovascular complications in East Asians with CKD warrants further investigation.

Vegetables and fruits are emphasized in the DASH diet; however, CKD dietary guidance often recommends lower intakes of certain fruits and vegetables in order to reduce potassium intake. The recommendation to reduce fruit and vegetable intake in CKD has been met with controversy, mainly due to the large amount of additional health benefits that come with eating a diet rich in fruits and vegetables. One observational study in Japanese pre-dialysis CKD patients found that higher NEAP, a risk factor for CKD progression, was associated with lower intakes of fruits and vegetables, and greater NEAP was associated with greater reductions in eGFR [71]. Fruits and vegetables are also high in fiber, which may be beneficial for patients with CKD. One longitudinal study in Chinese adults with CKD stages 3–4 found that increased fiber intake was associated with a smaller decrease in eGFR when compared to a lower fiber intake (<25 g/d), suggesting a higher fiber (>25 g/d) intake may slow the progression of CKD in this population [72]. While these studies provide preliminary evidence that consuming more fruits and vegetables may be beneficial in East Asian patients with CKD, more research is needed to fully explore the effects of a fruit- and vegetable-rich diet, such as the DASH diet, on CKD progression and complications in this population.

To our knowledge, there are no studies that have examined other DASH diet components, such as whole grains and low-fat dairy, in East Asian patients with CKD. These food groups are rich in potassium, phosphorus, and protein, three nutrients of concern in patients with CKD. Indeed, review papers from non-Asian countries have indicated that higher intakes of these nutrients may preclude recommending the DASH diet for patients with CKD if they have already experienced metabolic complications, or if their medication regimen increases their risk of metabolic complications [73]. However, whole grains are also important sources of fiber and magnesium, and low-fat dairy also provides magnesium as well as calcium. Excess calcium is a concern in CKD; however, both positive and negative calcium balance may lead to poor health outcomes in CKD patients (soft tissue calcification and loss of bone mineral, respectively), especially as it relates to CKD-mineral bone disorder (CKD-MBD) [74]. Additional studies, especially intervention studies, are needed to examine other DASH diet components in East Asians with CKD.

### 5.3. Comparing the DASH Diet with East Asian and International CKD Dietary Guidelines

Many countries, including China and Japan, have developed clinical guidelines for diagnosing, treating, and managing CKD based on the best available evidence. These guidelines include dietary recommendations for relevant nutrients including protein, lipids, carbohydrate, sodium, potassium, phosphorus, calcium, and fiber. CKD dietary guidelines from China [75], Japan [76], and the International Kidney Disease: Improving Global Outcomes (KDIGO) [77] guidelines, and the diet composition of the DASH diet [17,18] are summarized in Table 3. The DASH diet guideline for protein falls within the Acceptable Macronutrient Distribution Range set by the Institute of Medicine (now called the National Academy of Medicine), which also established a Recommended Dietary Allowance of 0.8 g/kg body weight/day [78]. This aligns with the recommendations for early-stage CKD from all three guidelines, which do not yet recommend reductions in protein intake. However, both Chinese and Japanese CKD dietary guidelines recommend reductions in protein intake for more advanced stages of CKD. For other macronutrients, the DASH diet guidelines align well with all three CKD dietary guidelines. Notably, besides protein, sodium is the only other nutrient with explicit intake recommendations in all three CKD dietary guidelines. The sodium recommendations of all three CKD dietary guidelines are similar, and the DASH diet guidelines for sodium fall well below these recommendations. Other nutrients of concern in CKD are potassium, phosphorus, and calcium. None of the CKD dietary guidelines provide explicit recommendations for potassium intake; both China and Japan provide recommendations based on serum potassium levels, and KDIGO states potassium intake recommendations should be individualized. The recommended potassium intake in the DASH diet is 4700 mg/d, which is higher than usual intakes in East Asia (see Table 1). In addition, phosphorus intake recommendations are inconsistent among the three CKD dietary guidelines: China recommends <800 mg/d, Japan recommends normal phosphorus intakes, and KDIGO recommends individualized intake guidelines. The DASH diet does not provide phosphorus intake guidelines, though some of the food groups recommended as part of the DASH diet are high in phosphorus. Calcium recommendations in CKD are also mixed: China recommends ≤2000 mg/d, Japan recommends normal calcium intakes, and KDIGO does not provide a recommendation for calcium. The DASH dietary guidelines recommend 1240 mg/d of calcium, which aligns with the CKD dietary guidelines from China. When comparing the DASH diet with CKD dietary guidelines in East Asia and globally, it seems the DASH diet could be recommended in early stages of CKD, based on individualized needs, with caution and careful monitoring for metabolic complications, a sentiment that has been echoed by others [73].

## 6. Discussion

The current review is a narrative review and not a systematic review, and is therefore limited in the conclusions that can be drawn from the summarized evidence. In addition, there are limitations in the available literature on the DASH diet and chronic disease in East Asian populations. Notably, more observational studies than intervention studies have been conducted in the East Asian population regarding the DASH diet and cardiometabolic health and CKD. A wider body of evidence on the DASH diet and chronic disease has come from research in Western countries, particularly the U.S., and the results are largely positive [79,80,81,82]. One could infer this relationship would hold true in East Asia, but additional studies are needed to confirm that the benefits of the DASH diet are truly translatable to this population. Cardiometabolic diseases as well as CKD are highly prevalent in East Asian countries. These diseases share many underlying risk factors, particularly elevated BP. It is well known that hypertension is a significant modifiable risk factor for CVD and CKD, and adequate BP control can considerably decrease the risk of cardiovascular events, diabetic nephropathy, and CKD progression. Diet is an important modifiable risk factor for these chronic diseases, and evidence indicates that adopting an overall healthy dietary pattern can be a valuable strategy to alleviate the burden of these diseases. Evidence in East Asian populations indicates that adopting a DASH diet may protect against cardiometabolic disease development and progression. Evidence suggests the DASH diet may also be protective against CKD development. When examining individual food groups and nutrients that are emphasized in the DASH dietary pattern, evidence from East Asian populations suggests that eating fruits and vegetables (which provide potassium, magnesium, and fiber), low-fat dairy products (which provide calcium, potassium, magnesium, and phosphorus), and lean protein (which also provides potassium, magnesium, and phosphorus) may help reduce the risk of developing CKD, but the benefits of these foods/nutrients in those who already have CKD is controversial and at odds with many of the typical food/nutrient restrictions recommended for CKD patients. The DASH diet limits sodium intake, and research in East Asian populations suggests that reducing sodium intake is beneficial both for preventing CKD and reducing CKD complications. Whether or not the DASH diet, or a modified version of the DASH diet, should be recommended for patients with CKD remains unclear. More research, especially randomized controlled trials, on the potential benefits of adopting a modified DASH diet in East Asian patients with CKD is needed. Additional evidence in populations who already have, or are at risk for, CKD and cardiometabolic diseases could help inform dietary guidelines as well as clinical practice in East Asian countries.

## Figures and Tables

**Table 1:** Comparison between nutrient intakes in the Dietary Approaches to Stop Hypertension (DASH) diet and usual nutrient intakes in selected East Asian countries.

Diet Component	DASH	China	Japan
Energy intake, kcal	2100 ^1^	2019	1913
Protein, % of energy	18	14.0	14.9
Fat, % of energy	27	36.5	25.0
Carbohydrate, % of energy	55	48.9	60.1
Sodium, mg	1150 ^2^	5925	4352
Potassium, mg	4700	1691	2372
Magnesium, mg	500	275	256
Calcium, mg	1240	436	522
Fiber, g	30	10.8	14.6

^1^ Reference range; actual kcal intake for each participant was based on individual needs and characteristics. ^2^ Level based on DASH-sodium trial. Abbreviations: DASH, Dietary Approaches to Stop Hypertension.

**Table 2:** Comparing the original DASH diet to the Japanese DASH diet.

Diet Component	Original DASH	DASH-JUMP (Japan)
Energy intake, kcal	2100 ^1^	1820 ^3^
Protein, % of energy	18	21
Fat, % of energy	27	18
Carbohydrate, % of energy	55	61
Sodium, mg	1150 ^2^	3057
Potassium, mg	4700	4333
Magnesium, mg	500	461
Calcium, mg	1240	1242
Fiber, g	30	28
Key Characteristics	Rich in: Fruits Vegetables Low-fat dairy Fish and lean meats Whole grains Nuts, legumes, seeds Reduced: Saturated fat, total fat, and cholesterol Red meat Sweets and sweetened beverages Refined grains	Rich in: Vegetables Low-fat dairy Whole grain brown rice Seaweed Mushrooms Reduced: Meat and eggs Sweets Oils and fats Pickles Other Features: Included one meal with soup per day Included typical Japanese dishes

^1^ Reference range; actual kcal intake for each participant was based on individual needs and characteristics. ^2^ Level based on DASH-sodium trial. ^3^ Also developed a 1650 kcal meal plan. Abbreviations: DASH, Dietary Approaches to Stop Hypertension; DASH-JUMP, DASH-Japan Ube Modified diet Plan.

**Table 3:** Comparison of the DASH diet to global and East Asian chronic kidney disease (CKD) dietary guidelines^1^.

		East Asian Dietary Guidelines by CKD Stage			International Guidelines by CKD Stage	
Dietary Component	DASH Dietary Pattern	CKD Stage	China	Japan	CKD Stage	Global (KDIGO)
Protein	18% of energy	G1–2	0.8–1.0 g/kg × SBW/d	0.8–1.0 g/kg × SBW/d	GFR <30 mL/min (G4–5)	0.8 g/kg/d
		G3–5 non-dialysis	0.6–0.8 g/kg × SBW/d	0.6–0.8 g/kg × SBW/d		
		G3–5 dialysis	1.0–1.2 g/kg × SBW/d			
Lipids	27% of energy	G1–5	25−35%	None	--	None
Carbohydrate	55% of energy	G1–5	55−65%	None	--	None
Sodium	11,150 ^1^	G1–5	<2000 mg/d	Salt 6 g/d (2400 mg of sodium)	G1–5	<2000 mg/day
Potassium	4700	G1–5	Limited when patient has hyperkalemia	4.0–5.4 mEq/L ^2^	--	Recommendation should be tailored to CKD severity and individual needs
Phosphorus	--	G1–5	<800 mg/d	Normal range	--	Recommendation should be tailored to CKD severity and individual needs
Calcium	1240	G1–5	≤2000 mg/d	Normal range	--	None
Fiber	30	G1–5	14 g/1000 kcal	None	--	None

^1^ Based on DASH-sodium trial. ^2^ Recommended serum levels. Abbreviations: CKD, chronic kidney disease; G, glomerular filtration rate category; SBW, standard body weight; KDIGO, Kidney Disease: Improving Global Outcomes.

## Supplementary Materials

The following are available online at https://www.mdpi.com/2072-6643/13/3/984/s1, Table S1: Summary of Studies Reviewed on the DASH Diet and Cardiometabolic Diseases and CKD1.
