# Supplementary material for: The DASH Diet and Cardiometabolic Health and Chronic Kidney Disease: A Narrative Review of the Evidence in East Asian Countries
Source: Nutrients. 2021 Mar 18;13(3):984. doi: 10.3390/nu13030984 (PMC8003274; doi:10.3390/nu13030984)
Supplement: Supplementary file 1 [file nutrients-13-00984-s001.pdf]

**Supplement**

**Table S1 Summary of Studies Reviewed on the DASH Diet and Cardiometabolic Diseases and CKD[1]**

| | First Author, year (reference number) | Study Population | Intervention/Exposure | Health Outcome Assessed | Overall Finding (Benefit, Harm, Neutral Effect) |
|---|---|---|---|---|---|
| **DASH Diet and CVD** | | | | | |
| Intervention studies | Kawamura, 2016 (40) | Japanese adults with elevated BP | Japanese-modified DASH diet | BP | Benefit |
| | Kim, 2013 (42) | Korean Americans with high BP | Korean-modified DASH diet education | BP | Benefit |
| | Lee, 2018 (43) | Korean adults with prehypertension or hypertension | Korean-modified DASH diet education | BP | Benefit |
| | Wong, 2015 (44) | Hong Kong patients with grade 1 hypertension | DASH diet education tailored to Chinese culture | BP and lipids | Neutral Effect |
| Prospective cohort Studies | Bai, 2017 (45) | Chinese adults free of chronic disease | DASH diet score | Risk of hypertension | Benefit |
| | Lin, 2013 (46) | Taiwanese adults | DASH diet score | Change in BP, risk of stroke | Benefit |
| | Neelakantan, 2018 (47) | Singapore Chinese adults free of CVD at baseline | DASH diet score | Risk of CVD mortality | Benefit |
| | Talaei, 2019 (48) | Singapore Chinese adults free of CVD at baseline | DASH diet score | Risk of coronary artery disease and stroke mortality | Benefit |

| | | | | | |
|---|---|---|---|---|---|
| Cross-sectional studies | -- | -- | -- | -- | -- |
| **DASH Diet and Type 2 Diabetes and Other Metabolic Disturbances** | | | | | |
| Intervention studies | Jin, 2020 (50) | Korean adults with type 2 diabetes | DASH-style diet plan | HbA1C levels | Benefit |
| | Choi, 2014 (54) | Korean elderly women with abdominal obesity | DASH diet education with n-3 supplements | Metabolic syndrome | Benefit |
| Prospective cohort Studies | Chen, 2018 (49) | Singaporean Chinese adults free of chronic disease | DASH diet score | Incidence of type 2 diabetes | Benefit |
| Cross-sectional studies | Kang, 2018 (52) | Korean postmenopausal women without diabetes | Korean-modified DASH diet score | Metabolic syndrome | Benefit |
| | Murakami, 2019 (53) | Japanese adults | DASH diet score | Metabolic risk factors | Benefit |
| | Gao, 2020 (55) | Chinese adults | DASH diet score | Hyperuricemia | Benefit |
| **DASH Diet and DASH Diet Components and CKD Risk** | | | | | |
| Intervention studies | Jardine, 2019 (60) | Chinese villagers | Sodium reduction education | Albuminuria | Benefit |

| | | | | | |
|---|---|---|---|---|---|
| Prospective cohort Studies | Yoon, 2017 (59) | Korean adults with normal kidney function with and without hypertension | Sodium intake | Risk of CKD | Harm (in hypertensive only) (lower intake=benefit) |
| | Mun, 2019 (61) | Korean adults with mildly impaired kidney function with and without hypertension | Potassium intake | Risk of CKD and change in eGFR | Benefit (in hypertensive only) |
| | Jhee, 2019 (63) | Korean adults | Fruit and vegetable intake | Risk of CKD and proteinuria | Benefit |
| | Lew, 2017 (65) | Singaporean Chinese adults | Red meat | Risk of ESKD | Harm (lower intake=benefit) |
| Cross-sectional studies | Lee, 2017 (56) | Korean elderly adults | DASH diet score and Korean-modified DASH diet score | Risk of CKD | Benefit |
| | Kim, 2018 (62) | Korean adults free of chronic disease | Mineral intake (Na, K, P) | Risk of CKD | Na—Neutral<br>K—Benefit<br>P—Benefit |
| | Higashiyama, 2010 (64) | Japanese adults | Protein intake | GFR and risk of CKD | Benefit |
| **DASH Diet Components in CKD** | | | | | |
| Intervention studies | Yu, 2012 (67) | Chinese adults with immunoglobulin A nephropathy | Sodium restriction | BP and proteinuria | Harm (lower intake=benefit) |
| | Sakaguchi, 2018 (71) | Japanese patients with stages 3-4 CKD | Magnesium supplementation | Coronary artery calcification | Benefit |

| | | | | | |
|---|---|---|---|---|---|
| Prospective cohort Studies | Kim, 2019 (69) | Korean adults with CKD stages 1-5 | Urinary potassium excretion | Decrease in eGFR or incident ESKD | Benefit (lower=harm) |
| | Koo, 2018 (70) | Korean adults with CKD stages 1-5 | Urinary sodium/potassium ratio | Decrease in eGFR or incident ESKD | Harm (lower ratio=benefit) |
| | Toba, 2019 (72) | Japanese patients with pre-dialysis CKD | Fruits and vegetables and NEAP (calculated from protein and potassium) | Change in eGFR | Benefit (>NEAP = <fruit and veg=harm) |
| | Lu, 2017 (73) | Chinese patients with stages 3-4 CKD | Fiber intake | Change in eGFR and CVD events | Benefit |
| Cross-sectional studies | Koo, 2014 (66) | Korean adult outpatient hospital patients with and without CKD | Urinary sodium excretion | BP control | Harm (lower=benefit) |
| | Yu, 2012 (67) | Chinese non-dialysis hypertensive CKD patients | Urinary sodium excretion | BP control | Harm (lower=benefit) |

[1]CVD, cardiovascular disease; BP, blood pressure; DASH, dietary approaches to stop hypertension; HbA1c, hemoglobin A1C; CKD, chronic kidney disease; ESKD, end-stage kidney disease; GFR, glomerular filtration rate; NEAP, net endogenous acid production.
